# Exposome and Immunity Training: How Pathogen Exposure Order Influences Innate Immune Cell Lineage Commitment and Function

**DOI:** 10.3390/ijms21228462

**Published:** 2020-11-11

**Authors:** Kevin Adams, K. Scott Weber, Steven M. Johnson

**Affiliations:** Department of Microbiology and Molecular Biology, Brigham Young University, Provo, UT 84602, USA; kevin.adams822@gmail.com

**Keywords:** exposome, trained immunity, epigenetics, NK cells, monocytes, macrophages, dendritic cells, histone modification, DNA methylation, COVID-19, SARS-CoV-2

## Abstract

Immune memory is a defining characteristic of adaptive immunity, but recent work has shown that the activation of innate immunity can also improve responsiveness in subsequent exposures. This has been coined “trained immunity” and diverges with the perception that the innate immune system is primitive, non-specific, and reacts to novel and recurrent antigen exposures similarly. The “exposome” is the cumulative exposures (diet, exercise, environmental exposure, vaccination, genetics, etc.) an individual has experienced and provides a mechanism for the establishment of immune training or immunotolerance. It is becoming increasingly clear that trained immunity constitutes a delicate balance between the dose, duration, and order of exposures. Upon innate stimuli, trained immunity or tolerance is shaped by epigenetic and metabolic changes that alter hematopoietic stem cell lineage commitment and responses to infection. Due to the immunomodulatory role of the exposome, understanding innate immune training is critical for understanding why some individuals exhibit protective phenotypes while closely related individuals may experience immunotolerant effects (e.g., the order of exposure can result in completely divergent immune responses). Research on the exposome and trained immunity may be leveraged to identify key factors for improving vaccination development, altering inflammatory disease development, and introducing potential new prophylactic treatments, especially for diseases such as COVID-19, which is currently a major health issue for the world. Furthermore, continued exposome research may prevent many deleterious effects caused by immunotolerance that frequently result in host morbidity or mortality.

## 1. Introduction

**Figure 1:**
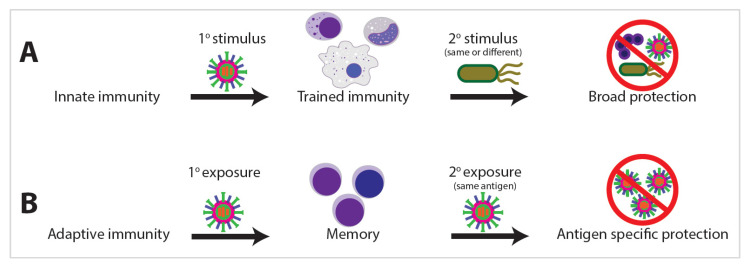
Innate immunity can result in broad immunological protection, whereas adaptive immunity provides antigen-specific protection. (**A**) For innate immunity, trained immunity can provide protection against heterologous stimuli, whereas with adaptive immunity (**B**), memory is elicited with primary exposure to a specific antigen, and then, a secondary exposure and subsequent protection requires exposure with the same antigen.

Innate immunity is the first line of host defense against external exposures. While traditionally viewed as primitive and nonspecific, a growing body of clinical and experimental evidence argues the innate immune system develops memory as a result of previous exposures, allowing the innate system to respond with enhanced and broad immunological protection upon exposure to a secondary stimulus [1,2]. This biological process of enhanced innate immunity response on secondary pathogen exposure has been termed “trained immunity” [2,3]. Trained immunity shares many phenotypic and epigenetic characteristics with adaptive immune memory; however, one of the starkest distinctions is the propensity for trained immunity to develop against heterologous stimuli (Figure 1). Innate memory is not antigen specific and is often protective against unrelated organisms, such as when vaccination for tuberculosis with Bacillus Calmette–Guerin (BCG) also affords protection against fungal or even viral infections such as SARS-CoV-2 [4,5,6].

**Figure 2:**
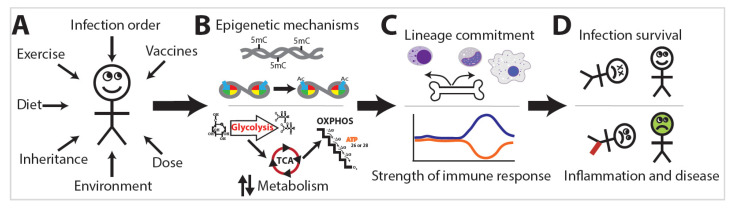
The effect of the exposome on innate immunity. (**A**) The order of exposure to external stimuli can have an important effect on innate immunity. (**B**) These stimuli elicit various epigenetic and metabolic changes that in turn dictate (**C**) lineage commitment and the strength of the innate immune response, which ultimately determine (**D**) the health outcome of the affected individual. The blue and orange lines in (C) denote innate immunity activation (blue line) versus tolerization (orange line).

The exposome is the compilation of everything an individual has encountered throughout their life [7]. Research into the exposome examines the combination of all exposures an individual has encountered during a set period of their life and the subsequent effects on that individual (Figure 2A) [7]. The exposome varies spatiotemporally, is highly dynamic and diverse, and provides a mechanism by which trained immunity or immunotolerance is established [8]. This occurs via distinct changes in leukocyte epigenetics and metabolism (Figure 2B) that initiate changes in leukocyte lineage commitment and the strength of the immune response (Figure 2C), eventually resulting in divergent levels of infection survival, inflammation, and disease (Figure 2D). Studies of the exposome are relatively sparse, yet the effects on human health are dramatic. Two major projects aimed at unraveling the exposome are The Human Early Life Exposome (HELIX) project and the EXPOsOMICS project. The HELIX project is a study aimed at measuring and correlating the effects of early-life exposures on human health [9]. The EXPOsOMICS project utilizes high-throughput sequencing and “omics” experiments to build models of lifetime exposures and their effects on human health [10,11].

Before an individual is born, they show evidence of epigenetic reprogramming because they are exposed to many stimuli encountered by their mother while also inheriting maternal epigenetic marks. Following their birth, individuals encounter environmental pollutants, pathogens, and allergens unique to them [12,13]. Even fluctuations in diet can cause long-term variations in health outcomes, variations that even exist between family members in the same household [13,14]. Furthermore, cohabiting individuals maintain uniquely identifiable external microbial clouds and internal microbiomes [12,15,16]. The combination of lifetime exposures establishes serious consequences in the development of inflammatory diseases [17,18].

Exposure to many airborne antigens can induce inflammatory airway diseases such as asthma. Individuals who walk along high-traffic streets experience airway acidification and immune cell infiltration, similar to that induced by intense exercise [19,20]. Unexpectedly, other airborne antigens such as dog-associated house dust can provide protection of the airways. While some of this protection is attributable to changes in the gut microbiome, the mechanism by which one person develops allergies to dog-associated dust while their sibling is protected remains unclear [21]. Because the training stimulus may be potentiated or abrogated by a secondary stimulus, it is reasonable to conclude that the order of exposures may actually be more important than the combination of exposures. This has been demonstrated in childhood vaccination as well as in animal models, many of which demonstrate significantly altered inflammatory profiles based solely upon the order of exposures received [22].

Monocytes/macrophages, dendritic cells (DCs), and natural killer (NK) cells are innate immune cells that all exhibit the ability to recollect a previous foreign encounter and subsequently mount an altered immunological memory response [23]. Exposure to high levels of bacterial lipopolysaccharide (LPS) and other toll-like receptor (TLR) agonists can induce a tolerogenic or “paralyzed” immune response, whereas BCG and many other pathogens induce a proinflammatory milieu of gene expression. This enhanced immune response is predicated upon extensive metabolic shifts in energy utilization and epigenetic regulation at the level of histone modification and differential DNA methylation. The metabolic and epigenetic effects of immune training can improve immune function, but they are also often maladaptive and result in arthritis, atherosclerosis, or allergies [1].

The metabolic and epigenetic changes observed are the result of the specific “training” the innate cells received upon the primary exposure. This, in conjunction with the various secondary stimuli encountered, results in the production of distinct cytokine profiles that are dependent on the order of pathogen exposure. Many studies have been conducted to understand the effects of environmental exposures on human health and immune function, emphasizing the total combination of exposures. However, they do not normally take exposure order into consideration, yet it is becoming clear that trained immunity is significantly influenced by exposure order. This review provides insight into cell lineage determination as well as epigenetic and metabolic mechanisms of trained immunity resulting from the exposome. Understanding not only the combinations of exposures but also their order is critical for implementing early health interventions, especially in low-birth-weight children, and is necessary to curb the rampant development of inflammatory diseases.

## 2. Influence of Pathogen Exposure on Innate Immune Cell Development and Function

### 2.1. Pathogen Exposure, Epigenetic Modifications, and Lineage Determination

During hematopoiesis, hematopoietic stem cells (HSCs) in the bone marrow undergo lineage-specific differentiation into either myeloid (myelopoiesis) or lymphoid (lymphopoiesis) progenitors. Lymphoid cell determination is accompanied by increased DNA methylation compared to myeloid cell determination. Consequently, inhibiting DNA methylation promotes myelopoiesis over lymphopoiesis [24]. Lineage determination is established by epigenetic changes in HSCs, which adapt to the exposures they encounter, ultimately passing previous encounters to the terminally differentiated daughter cells [25]. The epigenetic marks observed in trained HSCs are consequently found in both NK cells and monocytes [26]. This process is tightly regulated to prevent disease, as a dysregulated myelopoietic shift in HSCs is associated with immune-mediated diseases such as atherosclerosis and diabetes [27]. Myelopoiesis is inducible by various inflammatory stimuli and is a key factor in the establishment of long-term trained immunity, which can be perpetuated for months or years and even passed from mother to child [28,29]. While it has been shown that trained immunity can last up to a year or longer, exactly how long it lasts and whether innate memory can be refreshed or boosted to last longer is unknown and needs further study [30].

Infection of the bone marrow by pathogens can induce a myelopoietic shift. BCG induces the expansion of short-term HSCs and multipotent progenitors (MPPs) in trans by priming the HSCs to identify *mycobacterium tuberculosis* (MTB) [31]. Upon secondary stimulation with MTB, any myeloid progenitor cells derived from trained HSCs exhibit a memory immune response. This phenomenon is also observed during primary infection with *C. albicans*, which induces higher monocyte counts as well as protection against a subsequent lethal dose, in which the animals experience decreased fungal load [32]. Similarly, training with β-glucan mediates a favorable response to secondary challenge and protects the individual from chemotherapy-induced myelosuppression, offering a strong protective effect against myeloid cell depletion [33]. In fact, extended TLR2 agonist treatment also induces upregulated myelopoiesis, further indicating trained immune responses occur in HSCs, not just in terminally differentiated monocytes and macrophages [34].

Surprisingly, hypercholesterolemia and a high-fat diet (HFD) can also induce a myelopoietic shift in HSCs [27,35]. Cholesterol and other metabolites can induce DNA hypomethylation in the HSCs, which are pushed toward a myelopoietic fate [36]. In this way, obesity and adipose tissue transplants are both capable of generating increased myelopoiesis in wild-type (WT) mice [37]. Blood cell counts following an HFD or adipose tissue transplant reveal a significant increase in myeloid cell subsets in addition to an increased activation status for those circulating myeloid subsets, while HSCs exhibit an increased disposition toward myelopoiesis, with an accompanying transcriptional reprogramming of myeloid precursor cells [35,38]. This myelopoietic shift is regulated by ApoE, which may play a significant role in suppressing HSC and bone-marrow myeloid proliferation [39].

### 2.2. Discovery of Trained Immunity

**Figure 3:**
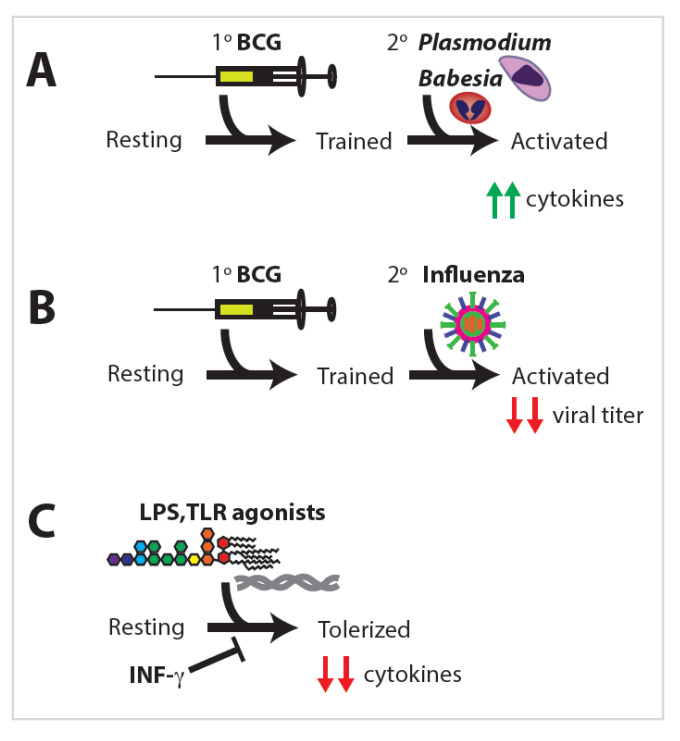
Innate immunity cells can become trained, activated, or tolerized depending on the primary and secondary stimuli. Stimulus with a prokaryotic antigen can cause trained cells that are capable of responding to (**A**) eukaryotic or (**B**) viral secondary stimuli. (**C**) Other stimuli can result in tolerization that can be blocked by pre-treatment with specific cytokines.

Trained immunity has shown a remarkable ability to provide protection against heterologous insults stemming from various primary exposures. Heterologous immunity was observed as early as 1976 when a group vaccinated mice with BCG and a prokaryote; challenged those mice with *Plasmodium* and *Babesia*, both eukaryotes; and observed that the mice vaccinated with BCG were protected against infection by both *Plasmodium* and *Babesia* in an antibody-independent manner (Figure 3A) [40].

**Figure 4:**
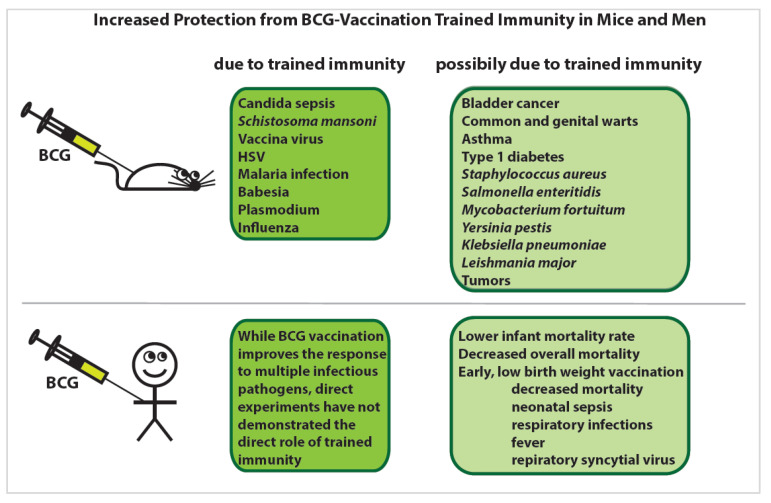
The proven and possible training effects of Bacillus Calmette–Guerin (BCG) vaccination. While BCG vaccination results in increased protection against a variety of pathogens due to trained immunity in both mice and humans (dark green boxes), the severity of multiple other diseases and pathologies is known to be abrogated by BCG vaccination, but this may be due to either innate trained immunity or lymphocyte-mediated heterologous effects (light green boxes).

Another group found that mice vaccinated with BCG exhibited decreased viral titers upon infection with influenza compared to unvaccinated mice (Figure 3B) [41]. This phenomenon was independent of adaptive immunity and showed that the innate immune system is capable of developing relatively long-lived memory. Since those initial studies, there has been much research into the extent to which innate immune training can elicit memory responses in NK cells, DCs, and monocytes/macrophages. Since the original discovery that BCG vaccination induces trained immunity, BGC has been shown to increase protection against viral, bacterial, fungal, parasitic, and even autoimmune diseases and tumors via trained immunity in mice and men [42] (Figure 4).

### 2.3. Natural Killer Cells

Natural killer cells are immune cells whose primary responsibility is the killing of virus-infected cells and tumor cells that do not properly display surface proteins. They share characteristics of innate immune cells (monocytes and DCs) and adaptive immune cells (B- and T-cells). NK cells respond to training stimuli by inducing epigenetic reprogramming and increased cytokine production, which resembles adaptive immune cell memory. Many of the same genes and remodelers are at work within the CD8+ T cell population and the NK cell population, at least during mouse cytomegalovirus (MCMV) infection [43].

Trained NK cells can be transferred from a sensitized mouse to a naïve mouse, and the naïve mouse will be protected. This is due to a Ly49C-I^+^ NK subpopulation localized in the donor’s liver [44]. Memory NK cells expand two- to three-fold in the presence of MCMV. The MCMV-trained cells maintain immunological memory for up to 37 days post-infection, at which point they return to pre-infection levels. Other training stimuli can induce longer or shorter periods of memory induction [45]. Not only can the NK cell memory response last for longer than one month, but it is also preserved through cell division [28]. These conclusions were drawn from studies performed in *Rag1* knockout mice stimulated with interleukin-12 (IL-12) and IL-18 to preclude the involvement of adaptive immune cells [28,46].

Upon encountering a secondary exposure, NK cells produce increased levels of IFN-γ and demonstrate increased cytotoxicity compared to naïve NK cells. This memory response is stimulated not only through MCMV infection, but also through BCG training and interleukin incubation [43,45,47,48]. BCG notably does not increase the number of NK cells in mice, yet when challenged with a lethal dose of the fungus *Candida albicans*, all mice survive, suggesting a role for NK cells in the protection conferred by BCG [48]. Similar results were observed when trained NK cells were challenged with *Toxoplasma gondii*, mycobacteria, leukemia cells, or MCMV. All these challenges resulted in elevated proinflammatory cytokine production, increased cytotoxicity, and improved survival [47,48,49,50].

### 2.4. Dendritic Cells

During an innate immune response, dendritic cells are responsible for phagocytosing the invading pathogen and presenting it to the adaptive immune system. DCs exhibit many functional programs similar to monocytes and macrophages and may be partially derived from a common precursor; however, DCs have also been shown to derive from lymphoid progenitors. This may indicate a niche in which DCs exhibit memory similar to both myeloid and lymphoid cells [51]. DCs can adopt proinflammatory or anti-inflammatory profiles based upon their exposure to various fungal and bacterial stimuli in the gut [52]. This presents a mechanism for immune tolerance and surveillance resulting from the diverse exposures encountered in the gut.

*C. albicans*, or its β-glucan cell wall, contributes to a tolerogenic immune state in DCs [52]. Similarly, severe sepsis induces immune tolerance, as indicated by a significant downregulation of proinflammatory cytokines [53]. In contrast to *C. albicans*-induced tolerance, encounters with *mycobacterium tuberculosis* (MTB) primes DCs toward a proinflammatory phenotype. The cells and cytokines induced by bacteria are protective against amoeba infection, as segmented filamentous bacteria induce increased IL-17a and IL-23 production, in addition to neutrophil and DC migration to the intestine [54]. Much of the pathogen-associated DC proinflammatory memory is reliant on NK cells in the bone marrow, which produce IFN-γ as in the case of *T. gondii* [49]. Interestingly, diet also plays a significant role in DC training. DCs isolated from mice fed a high-fat diet (HFD) exhibit significantly increased *Tnf-α*, *IL-6*, and *Nos2* gene expression. Upon secondary stimulation with LPS, HFD-fed DCs show further induction, significantly higher than that in normal diet-fed mice [38].

### 2.5. Monocytes and Macrophages

Monocytes/macrophages are the most extensively studied example of trained immunity, as they are inducible for both training and tolerance [55]. Upon traveling from the circulation into tissue, monocytes differentiate into tissue-resident macrophages, which perform the action of phagocytosing invading pathogens and presenting them to the adaptive immune system. Each individual TLR agonist elicits a different, unique response, which falls into two categories: tolerizing or training [56].

### 2.6. Macrophage Tolerance

Immune tolerance is a state of a diminished secondary immune response following a primary stimulation. Individuals who experience LPS toxic shock fall into a tolerogenic immune state that is unable to respond to secondary insults or infections. LPS and other TLR agonists can “overwhelm” the immune cells, driving the decreased expression of proinflammatory cytokines and altering TLR surface expression (Figure 3C) [55,57]. Interestingly, monocytes from premature infants also exhibit significantly fewer upregulated genes and lower expression of those genes when exposed to TLR agonists, particularly of cytokine genes and protein production, including IL-6, IL-1β, and TNF-α. Monocytes from premature infants additionally display lower levels of the phagocytosis of pathogens, which appears to be a requisite for cytokine production [58]. Even intense physical training can induce immune cell infiltration into the airways, as well as altered immune function that results in tolerance [20]. Immune tolerance does not only induce altered cytokine profiles; macrophages exposed to LPS also undergo metabolic shifts, switching from oxidative phosphorylation to glycolysis [59].

Macrophages induced by TLR stimulation express lower levels of inflammatory cytokines, with reactive oxygen species (ROS) production also diminished [60,61]. Specifically, exposure to LPS abrogates *IL-6* induction for 24 to 48 h [62], but if the histone mimic I-BET is used, macrophages are protected from becoming tolerized to LPS stimulation in the future, implicating specific histone modifications [63]. Significantly, tolerance is established in a dose-dependent manner, as exposure to a high dose of LPS induces tolerance to secondary LPS exposure; however, a low dose of LPS trains macrophages to respond strongly to secondary exposure [61].

Using a low dose of TLR agonist can prevent immune tolerance as can pre-incubation with inflammatory signals. The pre-treatment of cells with IFN-γ prevents the tolerization of primary human monocytes (Figure 3C) and restores the TLR4-mediated induction of various proinflammatory cytokines, including IL-6 and TNFα [64]. Some antimicrobial peptide genes also remain inducible despite LPS-induced tolerance, while non-tolerized genes respond to secondary stimulation much faster and to a higher degree than in naïve macrophages [62]. This implicates either the order or the combination of exposures in determining whether macrophages become primed or tolerized.

### 2.7. Macrophage Training

Macrophages have been used as an experimental model for a wide variety of training stimuli, and they are both dose and training/resting time dependent [65]. The first characterized training stimulus was BCG vaccination, which induces large-scale epigenetic reprogramming and significantly alters expression profiles [4]. BCG training induces the increased production of TNF-α and IL-1β two weeks and three months post-vaccination, and LPS-induced cytokine production remains significantly higher one-year post-vaccination [30]. Additionally, children vaccinated with BCG exhibit increased levels of cytokine production upon stimulation with purified protein injection even though immune cell counts are not significantly increased [66].

*C. albicans* and its β-glucan cell wall component are potent monocyte/macrophage training stimuli [57]. Priming with β-glucan results in increased cell viability in both mouse and human monocyte-derived macrophages [67]. As a result, monocyte counts in the bone marrow are increased following the low-virulence *C. albicans* infection of mice. When it was administered to mice prior to a lethal injection of *C. albicans*, the mice exhibited increased survival times and decreased fungal loads within organs [32,68]. In a separate study, mice were injected with a low dose of live *C. albicans*. Seven days later, the mice were challenged with a lethal dose, and the fungal loads, cytokine levels, and survival rates were measured. The WT mice were protected from the lethal dose as were RAG1-deficient mice, which lack T and B cells. However, CCR2-deficient mice, which lack monocytes, were not protected from the lethal dose following pre-infection. β-glucan, which makes up the cell wall of *C. albicans*, primes the production of proinflammatory cytokines in monocytes [69].

Macrophages also develop memory during an encounter with *Plasmodium falciparum*, the causative agent of malaria, which primes an LPS-inducible proinflammatory response in monocytes [56]. Children who exhibit a high level of IFN-γ upon monocyte stimulation with purified malarial proteins experience lower rates and severity of reinfection [70]. This may be similar to macrophages isolated from mice infected with a γ-herpesvirus. Macrophages from infected mice exhibited bactericidal activity, rapidly killing *L. monocytogenes* after uptake. Significant protection against *L. monocytogenes* was observed for up to three months [71]. This phenomenon requires live organisms, as heat-killed organisms do not typically produce such an effect [72].

Several inflammatory diseases are linked to inappropriate innate immune activation [18,27,73]. This is predominantly induced by oxidized low-density lipoprotein (oxLDL), though glucose and other metabolites also contribute [73,74,75]. OxLDL, but not LDL, is responsible for monocyte training in vivo, and while oxLDL does not promote cytokine production on its own, it does result in increased proinflammatory cytokine production upon restimulation [35,73]. Similarly, pre-incubation with glucose causes monocytes to adopt long-term memory. Monocytes pre-incubated with high levels of glucose exhibit a much stronger response to secondary stimulation compared to monocytes that are not pre-incubated [74]. Even fumarate induces trained immunity via epigenetic changes, promoting a similar profile to β-glucan stimulation [75], as does high levels of blood uric acid [76].

Monocyte/macrophage training is strongly associated with both epigenetic and metabolic changes. Trained cells exhibit increased glycolytic activity and altered oxidative phosphorylation due to changes in transcription factor (TF) binding [77]. This metabolic switch toward increased glycolysis upon training is dependent on both training and resting time [65]. Similarly, β-glucan stimulation induces a shift in metabolism toward glycolysis and oxygen consumption also increasing upon exposure to *C. albicans*; however, β-glucan alone induces a shift away from oxidative phosphorylation [78].

### 2.8. Granulocytes

Granulocytes are important innate immune cells but seem to play a minimal role in trained immunity based on the research conducted to date. They do play a supportive role in the development of trained immunity, as demonstrated by the loss of acquired resistance in mouse macrophages sensitized to the parasitic nematode *Nippostrongylus brasiliensis* in the absence of neutrophils. When neutrophils were depleted in primed mice, the protective effects of macrophage transfer to naïve recipients were abrogated [79].

## 3. Epigenetic Regulation of Trained Immunity

### 3.1. Epigenetic Regulation

Epigenetic regulation is the mechanism by which different cells and tissues in an organism perform cell-type-specific programs despite having virtually identical DNA in every cell. This is how each organ in the body establishes distinct phenotypic traits and is how trained immunity is regulated as well. Trained immunity is dictated by changes in chromatin accessibility due to differentially methylated DNA and changes in histone tail modifications, both of which result in chromatin remodeling. Chromatin remodeling via these modifications modulates the enhancement or repression of immune cytokine production [80]. Additionally, TF binding is a significant feature of epigenetic changes, lineage-specific activity, and cellular memory responding to environmental stimulation [81]. These three mechanisms work in concert to drive changes in cell fate and identity, as cells do not adopt new phenotypes or terminal differentiation without dynamic chromatin changes [82].

Chromatin accessibility and TF regulation are heavily influenced by external exposures. These external exposures can include microbes or their ligands, metabolites such as oxLDL, and ROS produced during exercise or inflammation [20,31,35]. Exposed cells can even influence neighboring cells, as observed when medium from exposed cells is transferred to nonexposed cells. The latter also exhibit altered cytokine production, indicating there are soluble molecules secreted from exposed cells that can alter the epigenetic programming of nonexposed HSCs [60]. This cascade of changes can be perpetuated across cell divisions and even from mother to child, despite the original stimulus being removed [28,29]. The heavy influence of the epigenome presents a mechanism by which innate immune training is passed down from mother to child to some degree [83].

The Human Early Life Exposome (HELIX) project is a study aimed at measuring and correlating the effects of early-life exposures on human health [9]. The EXPOsOMICS project utilizes high-throughput sequencing and “omics” experiments to build models of lifetime exposures and their effects on human health by measuring epigenetic, metabolic, lipidomic, and proteomic changes [10].

### 3.2. Histone Modifications

**Figure 5:**
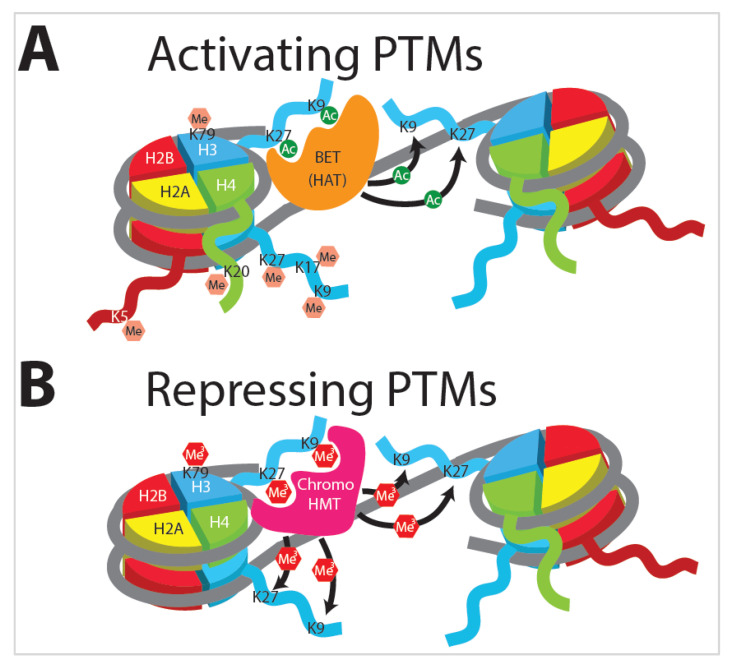
Transcriptional regulation is affected by histone protein post-translational modifications (PTMs). (**A**) Acetylation and monomethylation of specific histone-tail residues in nucleosomes results in activation of transcription. These marks can be transferred to adjacent nucleosomes by trans-acting proteins that bind to the modifications. (**B**) Trimethylation of specific histone residues results in repression of transcription. These repressive marks can in turn recruit other trans-acting proteins that transfer the repressive marks to neighboring nucleosomes.

Distinct cell types arise from virtually identical genetic material, thus implicating fundamental changes in gene expression and DNA accessibility in controlling cell fate and phenotype. Eukaryotic DNA is organized into nucleosomes, combinations of histone octamers and DNA. Histone octamers are composed of two copies each of H2A, H2B, H3, and H4 subunits, around which are wrapped ~147 bp of DNA (Figure 5). Each histone subunit has a free N-terminal tail, which can undergo enzymatically mediated post-translational modifications (PMTs). The addition of these moieties results in architectural changes to chromatin as well as transcriptional reprogramming. When the chromatin is condensed into heterochromatin, it becomes largely inaccessible to transcription, while loosening the chromatin into a euchromatic state either poises the DNA for transcription or allows genes encoded therein to be actively transcribed. Amid the vast repertoire of potential histone modifications, the two most extensively studied and broadly characterized are lysine acetylation (Figure 5A) and methylation (Figure 5B). These dynamic modifications are executed by various histone acetyltransferase (HAT) and histone methyltransferase (HMT) proteins [73,84].

Histone modification is crucial for virtually all instances of trained immunity, as the inhibition of the proteins involved in modification abrogates training. The nonspecific inhibition of histone methyltransferases with 5-deoxy-5-methylthioadenosine (MTA) or inhibition of histone acetyltransferases with epigallocatechin-3-gallate (EGCG) drastically inhibits the training of monocytes [57]. Additionally, combinations of various histone tail marks can epigenetically prime or poise enhancer and promoter elements for expression. These histone modifications respond rapidly to environmental stimuli, altering chromatin and gene expression profiles to adapt to injuries or pathogen insults, with significant epigenetic modification and remodeling observed between resting, tolerized, and trained immune cells [85,86].

### 3.3. Histone Acetylation

Histone acetylation by HAT proteins is generally found on histone 3 at lysines 9 (H3K9ac) and 27 (H3K27ac) (Figure 5A) and is generally associated with euchromatin and an enhanced transcriptional program [87]. The acetylation of lysine neutralizes the positive charge donated by the amino acid, thus decreasing the interaction with the negatively charged DNA backbone [88]. As the histone tail is loosened from the DNA, chromatin remodeling complexes with specialized bromodomains can bind to acetylated lysine tails and move or evict nucleosomes from promoter or enhancer regions [89]. Additionally, bromodomain proteins such as the bromodomain and extra-terminal (BET) domain-containing family of proteins have the ability to transfer the acetyl marks to neighboring nucleosomes (Figure 5A).

**Figure 6:**
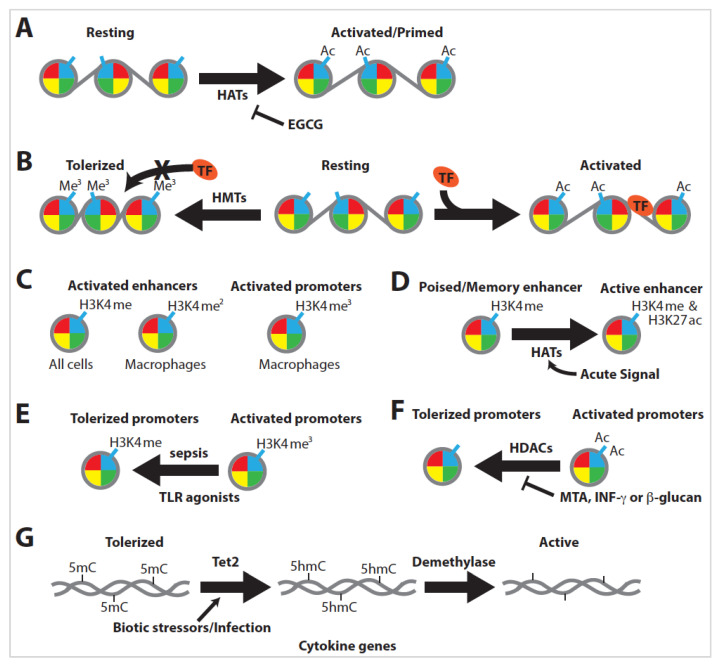
Chromatin can be compacted or decompacted, resulting in gene expression or repression, depending on the type of stimuli received by the cells and the resulting histone modifications or DNA methylation status. (**A**) Histone acetylation is required for innate immune training (e.g., inhibition of histone acetyltransferase (HAT) proteins via EGCG drastically inhibits the training of monocytes). (**B**) Methylation at lysine residues induces tighter DNA–protein interactions, which block transcription factor (TF) binding. (**C**) H3K4me1 is associated with enhancers in virtually all cells, H3K4me2 is associated with enhancers in macrophages, and H3K4me3 is associated with active promoters. (**D**) Genes associated with H3K4me1-bound enhancer sites trend toward low expression, whereas enhancer sites also bound with H3K27ac are upregulated. H3K27ac(−) enhancers are considered poised, while H3K27ac(+) enhancers are active. (**E**) Severe sepsis in mice causes a loss of H3K4me3 at tolerized genes. (**F**) Immune tolerance can be blocked by either the addition of MTA or proinflammatory signals such as IFN-γ or β-glucan, leading instead to inflammatory profiles. (**G**) DNA methylation can rapidly respond to infection, causing cytokine genes to be demethylated in response to biotic stressors.

BET governs the assembly of chromatin complexes at sites involved in inflammation by aiding in complex formation at acetylated histones (Figure 5A) [63]. Significantly, histone acetylation is required for innate immune training, as the inhibition of HAT proteins via EGCG drastically inhibits the training of monocytes (Figure 6A) [57].

### 3.4. Histone Methylation

Histone methylation is the addition of between one and three methyl groups to specific amino-acid residues on histone tails. Methylation at lysine residues induces tighter DNA–protein interactions, which block TF binding (Figure 6B). Additionally, chromodomain-containing proteins have the ability to transfer methyl marks to neighboring nucleosomes, allowing the silencing signal to be spread along the chromatin (Figure 5B). ChIP-seq analysis of histone tail marks shows differential epigenetic profiles between trained and non-trained immune cells and so can be used to identify the epigenetic state of a cell type [23,90]. Similarly, the ChIP-seq of the coactivator p300 is important for the identification of active compared to poised enhancers [91,92]. While methylation is generally associated with chromatin condensation and decreased gene expression, this is not always the case. The trimethylations of H3K27, H3K9, and H3K79 are linked to repression (Figure 5B), whereas the monomethylation of H3K27, H3K9, H4K20, H3K79, and H2BK5 is linked to gene activation (Figure 5A) [93,94,95,96,97].

Histone methylation is generally highly dynamic and can produce vastly different effects based on the location and the number of methyl marks [98,99]. H3K4me1 is associated with enhancers in virtually all cells, H3K4me2 is associated with enhancers in macrophages, and H3K4me3 is associated with active promoters (Figure 6C) [100,101].

### 3.5. Innate Immune Training Is Dependent on Histone Modification

Many latent enhancers are open to stimulation-specific transcription factors that can remain in place for an extended period of time. These latent enhancers become poised to respond quickly to a secondary exposure [4,101]. Genes associated with H3K4me1-bound enhancer sites trend toward low expression, whereas enhancer sites also bound with H3K27ac are upregulated. H3K27ac(−) enhancers are considered poised, while H3K27ac(+) enhancers are active (Figure 6D) [87,90]. This is significant in trained immunity, as prolonged exposure to training stimuli is unnecessary for long-term memory [87]. Moreover, the dose and nature of the training stimulus can produce opposing histone profiles [62].

Immune training with TLR agonists tends to generate an immunotolerant phenotype with accompanying histone modifications. Severe sepsis in mice causes a loss of H3K4me3 at tolerized genes (Figure 6E), resulting in a repressive state for histones at promoters of proinflammatory genes [53,62]. Coincident with differential histone methylation, histone deacetylases (HDACs) also contribute to immune tolerance [102]. However, immune tolerance can be blocked by either the addition of MTA or proinflammatory signals such as IFN-γ or β-glucan, leading instead to inflammatory profiles (Figure 6F) [5,64,85,103].

Upon encounters with pathogenic or inflammatory metabolic stimuli, innate immune cells undergo rapid epigenetic reprogramming at the level of histone modifications [69]. Monocytes trained with BCG or malaria exhibit an increase in H3K4me3 and a decrease in H3K9me3 at inflammatory cytokine promoters, which can last for weeks or months after the stimulus is removed [104,105,106]. Hyperglycemia, mevalonate, and oxLDL all similarly incite a strong proinflammatory response, with the enrichment of activating methyl and acetyl marks at H3K4 and H3K9 [26,73]. These training profiles are remarkably similar, indicating the same underlying mechanism of histone modification for innate immune training [23].

### 3.6. DNA Methylation

The most fundamental level of epigenetic regulation comes in the form of differential DNA methylation. DNA methylation is generally associated with decreased gene expression, while DNA demethylation, including that of the intermediate 5-hydroxymethylcytosine (5hmC), is associated with increased expression. Correspondingly, as genes experience demethylation, they also exhibit increased levels of activating histone marks [107]. These demethylation events are closely tied to immune cell activation and memory, from plants to humans. Furthermore, they are evolutionarily conserved in both adaptive and innate immunity [80,108]. DNA methylation can rapidly respond to infection, causing cytokine genes to be demethylated in response to biotic stressors (Figure 6G) [108]. Interestingly, infection-induced demethylation is almost exclusively found in distal enhancer elements, not at promoter regions. This active demethylation is associated with extensive epigenetic remodeling and is strongly predictive of changes in the expression levels of nearby genes [109].

Activating DNA demethylation is not coupled with DNA replication or cell division [80,107,110]. Under steady-state conditions, Tet proteins catalyze the conversion of 5-methylcytosine (5mC) to 5hmC, which can then be removed via demethylases (Figure 6G). Interestingly, 5hmC is associated with putative regulatory elements marking CG islands near the promoters of expressed cell-type specific genes, indicating that DNA demethylation is important for lineage-specific gene expression [111,112,113]. During inflammatory signaling, Tet2 is recruited to cytokine genes in response to cellular stimulation and is involved in regulating immune cell differentiation (Figure 6G) [110,112].

Differential DNA methylation can respond quickly to environmental exposures, resulting in dramatic transcriptional adaptation via DNA-binding factors and Tet proteins [110,114]. These demethylases and methyltransferases are inducible by metabolic components or infectious stimuli. Cholesterol blood lipid profiles positively correlate with global DNA methylation, with LDL cholesterol showing a strong, positive correlation [36]. Macrophages infected with live pathogens exhibit dramatic changes in DNA methylation patterns, whereas treatment with heat-killed organisms does not produce such an effect [72]. Ultimately, many inflammatory signals induce hypomethylation, promoting myelopoiesis over lymphopoiesis [24].

### 3.7. Transcription Factors and Enhancers

Following chromatin remodeling, many transcription factors are able to associate with the DNA to prime expression. Of particular importance are the TFs involved in cytokine production and those involved in the metabolic switching that accompanies immune cell activation, which can be assayed to determine cell fate and phenotype [81,84]. Inflammatory stimulation increases histone acetylation and p300 recruitment [103]. Inducible p300 binding is an efficacious target for retrieving macrophage-specific inflammatory enhancers [92,98]. Additionally, cell-type-specific factors can induce or restrict key transcription factor activity at both structurally and functionally distinct classes of enhancers. These cell-lineage-specific factors and general transcription factors can work in concert to prime enhancers throughout the genome to respond to specific stimuli [115].

Transcription factor binding can additionally create regions of low DNA methylation and may indicate a mechanism by which innate immune cells can induce DNA methylation or demethylation during training. This is the result of stimulus- and cell-specific transcription factor binding within immune cells or in HSCs [25,114]. Epigenetic changes are observed in several important signaling pathways due to pathogen-associated immune training [4,105,116]. Furthermore, metabolite-induced training is also strongly dependent on exposure-specific TFs such as MyD88 and mTOR [35,38,76].

Many genes related to glycolysis are significantly altered via HIF-1a, the master regulator of glycolytic gene expression in the cell [77,117]. Of note, the TFs of the STAT family are strongly associated with innate immune training. STAT1 induction is associated with active histone modification and is crucial for *Candida*-induced trained immunity [96,118]. Similarly, STAT4 is significant during the expansion of NK memory cell populations, which is also dependent on NKG2D and DNAM-1 [47,50].

### 3.8. Transcriptomics

Changes in gene expression levels are closely associated with changes in chromatin accessibility [55]. This process is intimately linked to chromatin marks and to specific exposures. The various histone tail marks and TFs discussed previously often directly correlate with transcription levels [90,93,94,95,119]. Measuring transcription levels in conjunction with mapping the chromatin landscape provides a more complete picture of the epigenetic mechanisms of trained immunity. As the chromatin relaxes, the DNA becomes more accessible to DNA-binding proteins such as TFs, which activate the expression of cell-type- or stimulus-specific genes [101,107]. In many cases, gene activation is maintained in the long term even after the initial stimulus has been removed, as with BCG in the bone marrow [30,31,87]. Expression does not always increase, but accessibility does, ultimately allowing for the rapid induction of proinflammatory gene expression upon a secondary insult [4,31]. This is commonly observed in HFD-fed mice, which show long-term transcriptomic reprogramming that is not reversed with short-term diet changes [35,38].

As innate immune cells become activated or tolerized, they develop distinct epigenetic profiles [85]. In some instances of immune training, those changes are highly correlated, as with β-glucan and mevalonate [23,85]. RNA-seq shows that cells injected with β-glucan demonstrate that pathways involved in innate immune function and pathways of cell metabolism, including glycolysis, cholesterol biosynthesis, and especially the mevalonate pathway, are overrepresented in the upregulated genes [33,69]. Consequently, upon secondary exposure to inflammatory signals, macrophages begin producing inflammatory cytokines and ROS in response to the perceived attack [29,49,120].

By contrast, tolerized cells adopt different epigenetic profiles recalcitrant to inflammatory stimulation, with an overrepresentation of anti-inflammatory gene expression and an underrepresentation of inflammatory cytokine expression [58,60,63]. Concordantly, pathways involved in lymphocyte development and function are overrepresented in the downregulated genes [33,69]. Remarkably, monocytes from premature infants exhibited significantly fewer upregulated genes and lower expression of those genes when exposed to inflammatory stimuli. This was particularly true of cytokine gene transcription and protein production [58]. In addition, genes associated with electron transport and glycolysis are downregulated in tolerized cells [77,78,117]. Together, the epigenetic landscape and transcriptomic profile of innate immune cells dictate the memory and activation of trained immune cells.

## 4. Exposome Alterations of Immune Function

### 4.1. Metabolism

Innate immune training is accompanied by dramatic shifts in cellular metabolism. Trained monocytes display high glucose consumption, lactate production, and NAD+/NADH ratio changes, reflecting a shift in the metabolism of trained innate cells. Human primary monocytes trained with β-glucan are primed to express increased levels of *HIF-1a* and subsequently *mTOR*, which induces a rapid shift toward glycolysis, accompanied by decreased oxygen consumption [78,121]. Conversely, *C. albicans* training induces a glycolytic shift, but oxygen consumption is dramatically increased [78]. BCG similarly induces monocytes to switch metabolism strongly toward glycolysis and other genes involved in metabolism and lipid biosynthesis; however, oxygen consumption also increases compared to that in non-trained monocytes [85,105].

Sterile exposures similarly generate epigenetic reprogramming in inflammatory pathways. Glucose metabolism and mTOR signaling are crucial in mevalonate-induced trained immunity as well as during fumarate accumulation [23,75]. LDL cholesterol is also a potent mediator of inflammatory signaling within innate immunity, resulting in proinflammatory cytokine production during secondary exposure [14,36]. Associated with obesity, specifically, high cholesterol, HSCs shift toward increased myelopoiesis and inflammatory cytokine production [27,37]. Uric acid levels can likewise induce mTOR activation, even in the absence of other inflammatory mediators [76].

Trained immunity is heavily dependent on a metabolic switch toward glycolysis through the TF HIF-1a, as it is the most readily available energy source [77,117,122]. Hyperglycemia, therefore, stimulates the immune system to adopt an inflammatory expression profile by stimulating increased glycolysis [26,74]. These effects play key roles in both the beneficial and maladaptive roles of trained immunity, with life-long consequences for human health [1,9].

### 4.2. Infection Survival

Trained immunity provides both the context and the mechanism by which exposure to an organism elicits improved survival rates in a T- and B-cell-independent manner. Even insects are able to mount trained immune protection against a subsequent lethal dose [123,124]. Likewise, the pre-treatment of mice with *C. albicans* has a remarkable ability to elicit decreased bacterial and fungal loads in the host, even when a normally lethal dose is administered [5,32,68,69,125]. This phenomenon is also observed following BCG vaccination, though remarkably, it provides protection against unrelated pathogens as well [126,127,128]. Individuals vaccinated with BCG experienced lower titers of parasites, bacteria, and fungi [129]. Most surprising is the ability of BCG vaccination to train the innate system against viruses [4,41,130].

Conversely, training with various viruses also primes the innate system against bacterial infection. Macrophages from MCMV-infected mice exhibit bactericidal activity, rapidly killing *L. monocytogenes* after uptake. Surprisingly, latently MCMV-infected mice are also resistant to *Y. pestis* [71]. Similarly, mice show improved resistance and pathogen clearance following pre-treatment with LPS, *C. albicans*, or various parasites [84,125,131]. This mechanism also protects individuals from malaria, as children who are exposed to purified malarial proteins experience lower rates and severity of reinfection resulting from high levels of proinflammatory cytokine production [70,132].

### 4.3. Inflammatory/Disease Phenotypes

While trained immunity can provide incredible benefits through the induction of heterologous protection, there is always the risk of inflammatory disease. Environmental factors, specifically in terms of the exposome, are important for the development of diseases such as atherosclerosis and other diseases due to the propensity of trained monocyte-derived macrophages to infiltrate tissue [18,27,38]. Obesity and hypercholesterolemia are associated with increased myelopoiesis, which induces trained macrophages to release proinflammatory cytokines in response to sterile signals [27,35,37]. This leads to a high uptake of oxLDL and an inappropriate inflammatory environment that damages host tissue [73].

Environmental exposures can be similarly destructive, particularly in the gut or lungs. Intense exercise and air pollution negatively affect air capacity and can cause airway acidification and innate cell infiltration [19,20]. As a result, individuals are more susceptible to reactive airway disease, asthma, or infection resulting from altered physiological function [19,23]. Similarly, improper inflammation can cause diseases in the gut ranging from Crohn’s disease to cancer [65,133,134].

### 4.4. Heterologous Immune Protection

What makes trained immunity distinct from adaptive memory is its ability to consistently respond to heterologous environmental exposures [22,116]. Unlike the adaptive immune system, which generally confers antigen-specific protection, innate immune training coordinates a general inflammatory state that can respond strongly to non-specific targets (Figure 1) [134,135,136]. It is predictable that BCG would protect against MTB, yet it consistently demonstrates training against unrelated bacteria such as *Staphylococcus aureus* or *Escherichia coli* [30]; viruses, including some strains of influenza [41,137,138], some herpesviruses [71,130], and even Yellow Fever [4]; many eukaryotic parasites ranging from *Plasmodium* to *Babesia* [40,48]; and even cancer [133].

Microbes found within the guts of individuals act as strong trainers of innate immunity. Segmented filamentous bacteria can cause immune cell migration to the intestine and stimulate protection against amoeba infection [54]. Similarly, *C. albicans* in the gut contributes to a tolerogenic immune state that protects the host from excessive or destructive inflammation [52,125]. Conversely, exposure to environmental stimuli trains the innate system in opposition to the induction of a tolerogenic state [29,67,120,139]. Training with several eukaryotic parasites encourages a proinflammatory profile against many bacterial, fungal, and parasitic microbes [56,131]. Interestingly, when individuals encounter multiple exposures, they display unique expression programs most strongly influenced by the latest contact [22].

### 4.5. Sex-Dependent Differences

It is important to be aware of sex-associated differences in immunity when studying vaccine development or immunostimulatory therapies [83]. In the case of diphtheria, pertussis, and tetanus (DPT) vaccination, infant mortality is significantly increased, particularly among female recipients [134]. This is in contrast to the sex-dependent benefits of BCG and the measles vaccine (MV), in which females have lower mortality rates than males [126]. Interestingly, the sex-associated differences with BCG are more pronounced in males early on but wane quickly. Females exhibit changes later and for a longer period. Furthermore, other live-attenuated vaccines show greater benefits in females, whereas the detrimental effects of non-live vaccines are more exaggerated [127,140]. These sex-dependent differences cannot be discounted when studying trained immunity or exposomics, as the effects of exposures may be beneficial in one sex while detrimental in the other.

### 4.6. Trained Immunity Mechanisms

Innate immune training is coordinated by exposure-specific epigenetic changes that induce altered transcriptomic profiles. Innate cells encounter either sterile or pathogenic stimuli, which generate distinct inflammatory profiles in a T-cell-independent manner [71]. Stimulation with pathogens often primes immune cells to release stimulatory cytokines, which in turn train other cells [49,106]. However, this phenomenon is highly dependent on local immunity [41]. In the gut, exposures to pathogens tend to induce immune tolerance locally, while inflammatory training in response to the same pathogen can still occur elsewhere in the host [141,142]. Of particular note is when stimuli are found within the circulatory system. Many sterile inflammatory mediators such as glucose and cholesterol can induce organism-wide changes [142]. This provides a mechanism by which environmental exposures can establish trained immunity locally or globally.

Innate immune cells can be sensitized by cytokines produced either by themselves or by other white blood cells, causing the chromatin to relax and adopt acetylated histones at promoter and enhancer regions [65]. Pre-treatment with inflammatory cytokines prevents TLR-inducible tolerization in NK cells and macrophages [64,118]. Enhancer landscapes can be primed by other environmental cues or developmental programs, both of which lead to rapid and flexible responses to stimuli and differentiation [92,115]. During this training, H3K9me may be reduced at key inflammatory cytokine genes, allowing for a more rapid secondary immune response upon restimulation [96,99]. This provides a mechanism by which memory is established and maintained via cytokine exposures.

During the course of infection, pathogenic components are frequently shuttled to the bone marrow, where they train HSCs. Upon primary encounters, HSCs undergo extensive differential DNA methylation due to transcription factor binding, particularly near the promoters of expressed, cell-type-specific genes [113,114]. Demethylation is mediated by Tet proteins, which catalyze the conversion of 5mC to 5hmC, which is subsequently removed by demethylases (Figure 6G) [110,112]. Interestingly, 5hmC itself acts to prime gene expression at cell-type-specific genes [111,112,113]. However, training does not necessarily occur in the bone marrow or blood but may be perpetuated in the tissue [143]. This indicates a mechanism by which immune training can be maintained far longer than the life of any individual cell population.

Upon secondary exposure, trained innate cells respond more quickly and more aggressively than during primary stimulation [103]. This immune cell activation is associated with significant metabolic reprogramming through HIF-1a [59,77,117]. Other pathogen surface markers can likewise promote metabolic switching via TLR stimulation [78]. By shifting their metabolism toward higher glycolytic activity, cells ramp up the production of cytokines and ROS involved in pathogen clearance [65,77]. This is dependent on the TF STAT1, as individuals with mutated STAT1 are unable to mount trained immune responses [118]. This provides a mechanism by which immune training can be detected by measuring cellular metabolism.

## 5. Conclusions

**Figure 7:**
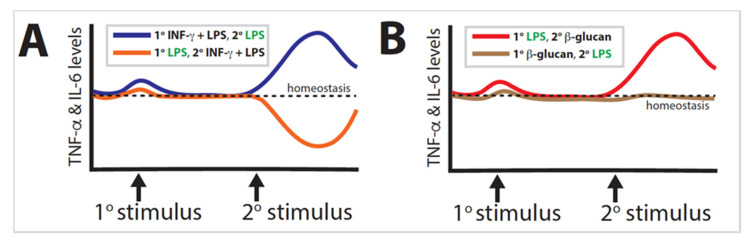
Inverting the order of primary and secondary stimuli can result in vastly different innate immunity outcomes. (**A**) Changing the order of stimulus with INF-γ + lipopolysaccharide (LPS) versus stimulus with LPS alone can result in innate immunity activation (blue line) versus tolerization (orange line). (**B**) Similar results can be seen by inverting the order of stimulation by LPS versus β-glucan resulting in activation (red line) versus perpetuation of the homeostatic state (brown line).

Trained immunity is a well-established phenomenon found in organisms ranging from plants to humans. Much of the research performed has focused solely on either the primary or the secondary stimulus, illustrating the extent to which the combination of exposures determines epigenetic reprogramming; however, in many cases, the order of exposures is likely more significant than the combination of stimuli can adequately explain. The most dramatic example is when primary LPS and secondary LPS + IFN-γ are inverted (Figure 7A). A similar effect is observed when primary LPS and secondary β-glucan are likewise inverted (Figure 7B). These examples demonstrate a proclivity for the secondary exposure to induce a stronger influence than the primary exposure.

Further research conducted on the exposome should take order into consideration, as an emphasis on solely one stimulus paints an incomplete picture of immune training. This may be particularly substantial in airway diseases, such as asthma or allergies, wherein some individuals develop allergies to pollen while others do not [20]. This may be partially attributable to whether the individual’s immune system was primed prior to exposure to said allergen. Understanding exposure order could significantly benefit atopic individuals.

It is worth reiterating that vaccine order can dramatically affect mortality rates in newborns [22]. Accordingly, vaccination schedules should be reevaluated, as specific vaccines can induce either heterologous immune protection or innate immune tolerance. By reordering vaccine administration, physicians can potentially reduce childhood sepsis and airway infections without necessitating additional vaccine development. Animal studies and, eventually, human trials may be beneficial in reducing childhood mortality, thus reducing global healthcare costs through the non-specific prevention of infections.

Further vaccine development must also take into consideration heterologous innate immune effects. While trained immunity presents a compelling avenue for novel vaccine development, care must be taken to prevent detrimental immune tolerance. Further research should be conducted to better understand exposure order. Inverting BCG vaccination with its various secondary exposures would be an important first step, as it represents an existing vaccine currently in deployment with potential implications for infectious agents such as SARS-CoV-2 [6]. In fact, extensive research is currently underway examining the potential protective effects that previous BCG vaccination and its subsequent innate immune memory may have on preventing COVID-19 or at least in decreasing the severity of the disease [144]. It is hypothesized that since BCG vaccination can induce trained immunity against heterologous infections, including respiratory viral infections, it could offer a measure of protection against SARS-CoV-2, effecting a quicker innate immune response to initial infection, which would decrease the viral load until the adaptive immune response can kick in. Since immune tolerance is a possible outcome of the innate training, controlled clinical studies need to be conducted to make sure that the initial exposure does not inhibit the innate response, increase early viral levels, and then cause an over-stimulation of the adaptive immune response, which is common in serious COVID-19 cases. While innate training represents a powerful option for combating multiple pathogenic threats, these hypotheses need further rigorous clinical study before therapeutic conclusions and recommendations can be made. By altering the administration of primary and secondary exposures, researchers will better explain the extent to which exposure order can reprogram the innate immune system. Ultimately, it is not the individual components that dictate the outcome of the innate immune response, but the combination, order, and potency of the stimuli decide the fate of the system and even the survival of the host organism.

## Abbreviations

BCG	Bacillus Calmette–Guerin
HELIX	The Human Early Life Exposome
DC	Dendritic cell
NK	Natural killer cells
LPS	Lipopolysaccharide
TLR	Toll-like receptor
HSC	Hematopoietic stem cells
MPP	Multipotent progenitors
MTB	*Mycobacterium tuberculosis*
HFD	High-fat diet
MCMV	Mouse cytomegalovirus
IL	Interleukin
oxLDL	Oxidized low-density lipoprotein
HAT	Histone acetyltransferase
HMT	Histone methyltransferase
MTA	Methylthioadenosine
EGCG	Epigallocatechin-3-gallate
ROS	Reactive oxygen species
TF	Transcription factor
DPT	Diphtheria, pertussis, and tetanus
MV	Measles vaccine
5mC	5-methylcytosine
5hmc	5-hydroxymethylcytosine
SARS-CoV-2	Severe acute respiratory syndrome coronavirus 2
COVID-19	Coronavirus Disease 2019
